# Structural Characterizations and Dielectric Properties of Sphere- and Rod-Like PbTiO_3_ Powders Synthesized via Molten Salt Synthesis

**DOI:** 10.1186/s11671-019-2899-9

**Published:** 2019-02-21

**Authors:** Qing Ji, Piaojie Xue, Heng Wu, Zhipeng Pei, Xinhua Zhu

**Affiliations:** National Laboratory of Solid State Microstructures, School of Physics, Nanjing University, Nanjing, 210093 China

**Keywords:** PbTiO3 powders, (Template) molten salt synthesis, Dielectric properties, Microstructural characterization

## Abstract

By reaction of PbC_2_O_4_ and TiO_2_ in the eutectic NaCl-KCl salts, both sphere- and rod-like PbTiO_3_ (PTO) powders were synthesized via molten salt synthesis (MSS) and template MSS methods, respectively. X-ray diffraction patterns reveal that all the PTO powders crystallize in a tetragonal phase structure. Increasing the molar ratio of PbC_2_O_4_:TiO_2_:NaCl:KCl from 1:1:10:10 to 1:1:60:60 in the MSS process has little effect on the sphere-like morphology of the PTO powders synthesized at 950 °C for 5 h. Large-scale polycrystalline rod-like PTO powders with diameters of 480 nm–1.50 μm and lengths up to 10 μm were synthesized at 800 °C for 5 h by template MSS method, where the rod-like anatase TiO_2_ precursors were used as templates and the molar ratio of PbC_2_O_4_:TiO_2_:NaCl:KCl was equal to 1:1:60:60. X-ray energy dispersive spectroscopy spectra reveal that all the PTO powders are composed of Pb, Ti, and O elements, and the measured Pb:Ti atomic ratios are close to 1:1. In the template MSS process, the molten salt content plays an important role in forming the rod-like PTO powders. Under low molten salt content, the rod-like PTO powders cannot be synthesized even if the rod-like TiO_2_ templates are used. In addition, prolonging the reaction time suppressed the formation of rod-like PTO powders but promoted the formation of sphere-like PTO nanoparticles. The dielectric properties the sphere- and rod-like PTO powders were comparatively investigated. At room temperature, the dielectric constant and dielectric loss of the spherical PTO powders synthesized by MSS method with the molar ratio of PbC_2_O_4_:TiO_2_:NaCl:KCl equal to 1:1:30:30 were ~ 340 and 0.06 (measured at 10^6^ Hz), respectively. The corresponding values for the rod-like PTO powders synthesized by template MSS method with the molar ratio of PbC_2_O_4_:TiO_2_:NaCl:KCl equal to 1:1:60:60 were 140 and 0.08, respectively. The present results demonstrate the sphere-like PTO powders have better dielectric properties, which have promising applications in the fields of multilayer capacitors and resonators.

## Introduction

Perovskite oxides with the general formula ABO_3_ are one of the most important classes of materials in solid-state chemistry, which have been widely used in the fields of ferroelectricity, magnetism, optoelectronics, and energy conversion [1–3]. Among all the members of the perovskite oxide family, PbTiO_3_ (PTO) has the highest tetragonal distortion (*c*/*a* ~ 1.064), which makes it remarkable over others. This large tetragonal distortion corresponds to the highest spontaneous polarization among all the ferroelectric perovskite oxides. As a paradigm of perovskite ferroelectric oxides, PTO possesses excellent dielectric, piezoelectric, and pyroelectric properties [4, 5]. However, pure PTO ceramics are difficult to be prepared as high-density and monolithic ceramics due to the problems such as the thermal expansion mismatch, mechanical stretching force, and microcracks in the PTO ceramics. In the past decade, much work has been devoted to the synthesis of PTO powders by various routes, such as solid-state reaction [6], the sol-gel process [7, 8], the hydrothermal method [9, 10], the Pechini method [11], co-precipitation [12], and so on. However, in all these methods, calcination at more or less high temperature is required to get pure crystallized ferroelectric PTO. Unfortunately, high-temperature calcination usually produces agglomerated powders with a coarse particle size which requires additional milling process. Contamination and other undesirable features during the milling process can create defects in the manufactured products, damaging the electrical properties of the PTO ceramics.

Molten salt synthesis (MSS) method is an effective way to prepare perovskite oxide electronic ceramic powders, which involves molten salt used as the medium for synthesizing pure perovskite oxides from their constituent materials (oxides and carbonates) at a relatively low temperature and in a shorter reaction time as compared with the conventional solid-state reactions [13]. Recently, perovskite PTO powders are synthesized by molten flux reaction using NaCl and NaCl-KCl as the reaction media [14–16]. The formation of spherical PTO powders was achieved by the dissolved PbO diffusing onto the TiO_2_ surface in the molten salts and reacting in-situ to form PTO nanoparticles, and then following the nucleation and growth of PTO nanoparticles. As compared with the PTO powders, the synthesis of PTO 1D nanomaterials (e.g., nanorods, nanowires, and nanotubes) by MSS method has lingered far behind. The main reason is due to the challenges in synthesizing high-quality PTO 1D nanomaterials because the high symmetry of the perovskite structure can easily lead to PTO growing into a cubic block. To date, only a few works about the synthesis of PTO 1D nanomaterials by MSS method are available in the literature. Deng et al. [17] synthesized PTO nanorods with diameters of 50–80 nm and lengths of a few micrometers at 700 °C by using a surfactant (polyoxyethylene (9) nonylphenyl ether, NP-9)-assisted approach in a NaCl molten salt medium. The growth of the PTO nanorods was attributed to the dispersion of fine PTO nanoparticles and their re-deposition on larger particles, leading to the formation of nanorods along the axial direction under the combination effects of NP-9 surfactant and flux of NaCl. Cai et al. [15] reported on the synthesis of single-crystalline PTO nanorods with diameters of 0.1–1.0 μm and lengths of up to a few micrometers by template MSS method, where NP-9 was used as a surfactant and the rod-shaped TiO_2_ precursors were used as the templates for titanium sources. The size and morphology of the rod-shaped TiO_2_ templates were retained in the synthesized PTO particles. Similarly, needle-like PTO powders were also synthesized via template MSS method, where pure needle-like TiO_2_ particles were used as the templates [18]. The needle-like PTO particles synthesized by template MSS method at 800 °C had a length of 30–100 μm and diameters of 500 nm–2.0 μm.

Despite the above reports on the synthesis of PTO nanomaterials by MSS method and template MSS method, there are scarce data on the dielectric properties of the PTO powders. In addition, the formation mechanism of PTO nanorods by template MSS method is not well understood. In this work, we report on the synthesis of sphere- and rod-like PTO powders via (template) MSS methods by reaction of PbC_2_O_4_ and TiO_2_ in the eutectic NaCl-KCl salts without using the NP-9 surfactant. The influence of the processing parameters of the template MSS method such as the reaction time and molten salt content on the formation of rod-like PTO powders was investigated in detail. The results demonstrate that the molten salt content plays a critical role in forming the rod-like ABO_3_ compounds with cubic or pseudo-cubic crystal structure in the template MSS process. At low molten salt content, the PTO nanorods cannot be synthesized even though the rod-like TiO_2_ templates are used in the template MSS process. The dielectric properties of sphere- and rod-like PTO powders synthesized by MSS method and template MSS method were also comparatively studied, and the results demonstrated the sphere-like PTO powders exhibited better dielectric properties.

## Methods

### Materials

Analytical grade reagents of PbC_2_O_4_ and TiO_2_ (with sphere-like morphology and mixed phase structure of anatase and rutile) were purchased from Sigma-Aldrich (Shanghai) Trading Co., Ltd. Analytical grade reagents of NaCl and KCl salts, K_2_CO_3_, AgNO_3,_ and HCl solutions were obtained from Shanghai Chemical Reagent Co., Ltd.

### Synthesis of Sphere-Like PTO Powders by MSS Method

Sphere-like PTO powders were synthesized via MSS method by reaction of PbC_2_O_4_ and TiO_2_ in the eutectic NaCl-KCl salts. The molar ratios of PbC_2_O_4_:TiO_2_:NaCl:KCl were selected as 1:1:10:10, 1:1:20:20, 1:1:30:30, 1:1:40:40, and 1:1:60:60. The mixtures of PbC_2_O_4_, TiO_2_, NaCl, and KCl were ground in a mortar and pestle for 30 min and then heated in the alumina crucibles to 950 °C for 5 h. Finally, the products were cooled naturally in the furnace to room temperature, and they were washed for several times with deionized water until no free chloride ions were detected by AgNO_3_ solution to ensure complete removal of the salts. After washing, the products were dried at 120 °C for 4 h for structural characterizations.

### Synthesis of Rod-Like PTO Powders by Template MSS Method

Rod-like PTO powders were synthesized via template MSS method, where the rod-like anatase TiO_2_ particles were used as the titanium source. The rod-like TiO_2_ templates were prepared from the rod-shaped K_2_Ti_4_O_9_, following a procedure reported previously by Hayashi et al. [19]. First, K_2_CO_3_ oxide was mixed with TiO_2_ oxide with a molar ratio of 1:3, and then the mixture was heated at 1000 °C and kept for 18 h. Finally, the product was cooled naturally in the furnace to room temperature and washed for several times with deionized water to remove residual K_2_CO_3_. The obtained main product of K_2_Ti_4_O_9_ was washed in 70 °C HCl solution (concentration of 1 M) for 2 h to extract K_2_O, and the resultant phase was TiO_2_·nH_2_O, which was annealed for 1 h at 600 °C, 700 °C, and 800 °C, respectively, to obtain the rod-like TiO_2_ compounds. And then, PbC_2_O_4_ was mixed with rod-like TiO_2_ templates and NaCl-KCl molten salt with molar ratios of PbC_2_O_4_:TiO_2_ (templates):NaCl:KCl equal to 1:1:20:20 and 1:1:60:60, respectively. The two mixtures were annealed at 800 °C for different hours (e.g., 1 h, 5 h, and 10 h). The final products were washed and dried in similar steps above.

### Microstructural Characterization

The phase structures of the as-prepared PTO powders were characterized by X-ray powder diffraction (Rigaku D/Max-RA, Cu Kα radiation). A step size was 0.01° per second, and the 2θ range was 15–70°. The surface morphologies of the PTO products were examined using scanning electron microscopy (SEM, Hitachi S-3400 N II, 30 kV) fitted with an X-ray energy-dispersive spectroscopy (EDS) (EX-250 spectroscopy, HORIBA Corporation) for element detection. The SEM samples were prepared by sprinkling powder on carbon tape and thereafter coated with gold if necessary.

### Dielectric Measurements

For the dielectric properties measurements, the dried sphere- and rod-like PTO powders were pressed into pellets of 12 mm in diameter and 1.0 mm thickness under a pressure of 7 MPa, which were annealed at 1150 °C for 2 h in air. Subsequently, the surfaces of annealed pellets were ground and polished followed by painting silver paste on both surfaces. The silver pastes were then fired at 550 °C for 60 min. The dielectric constants and the dielectric losses of the annealed PTO powders were measured at room temperature from 10 Hz to 1 MHz by using an Agilent 4192 A impedance-analyzer.

## Results and Discussion

### Phase Structure and Morphology of PTO Powders Synthesized by MSS Method

**Fig. 1:**
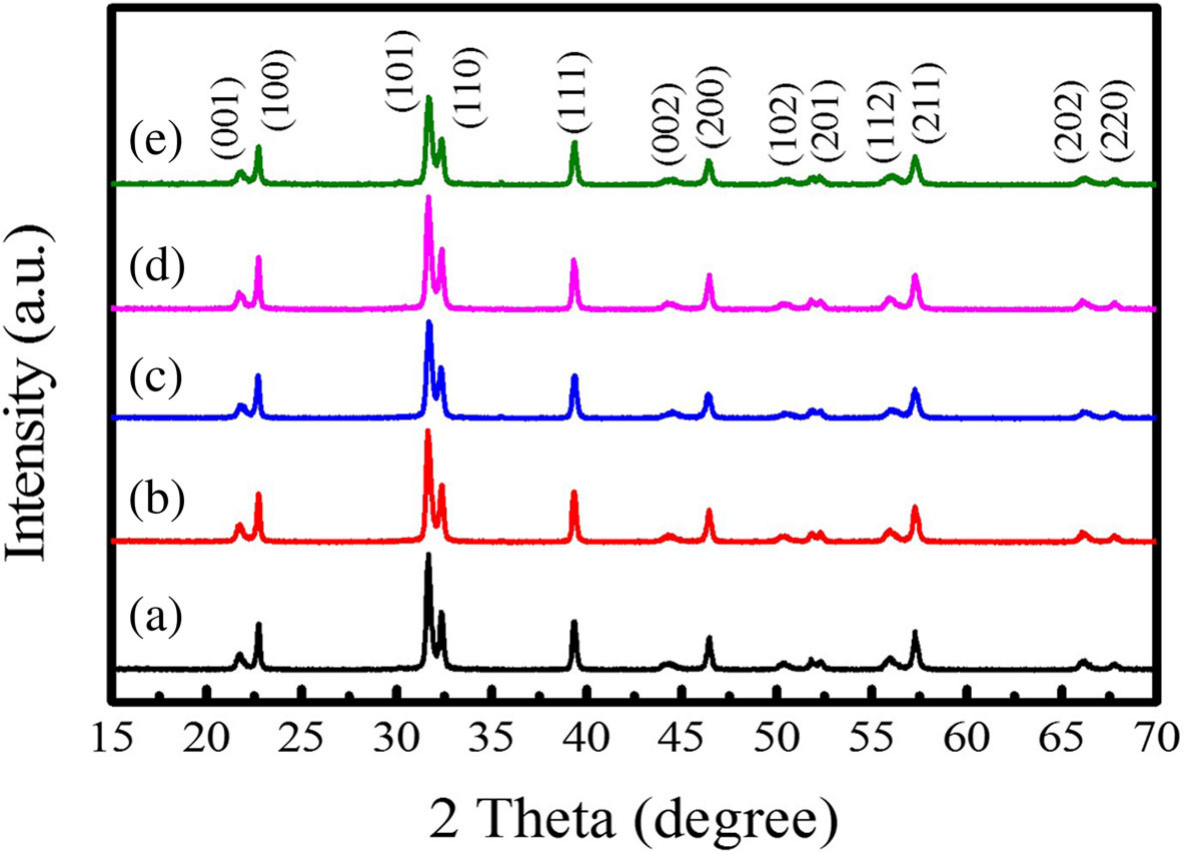
XRD patterns of the sphere-like PTO products synthesized by MSS method at 950 °C for 5 h with the molar ratios of PbC_2_O_4_:TiO_2_:NaCl:KCl equal to (a) 1:1:10:10, (b) 1:1:20:20, (c) 1:1:30:30, (d) 1:1:40:40, and (e) 1:1:60:60, respectively

**Fig. 2:**
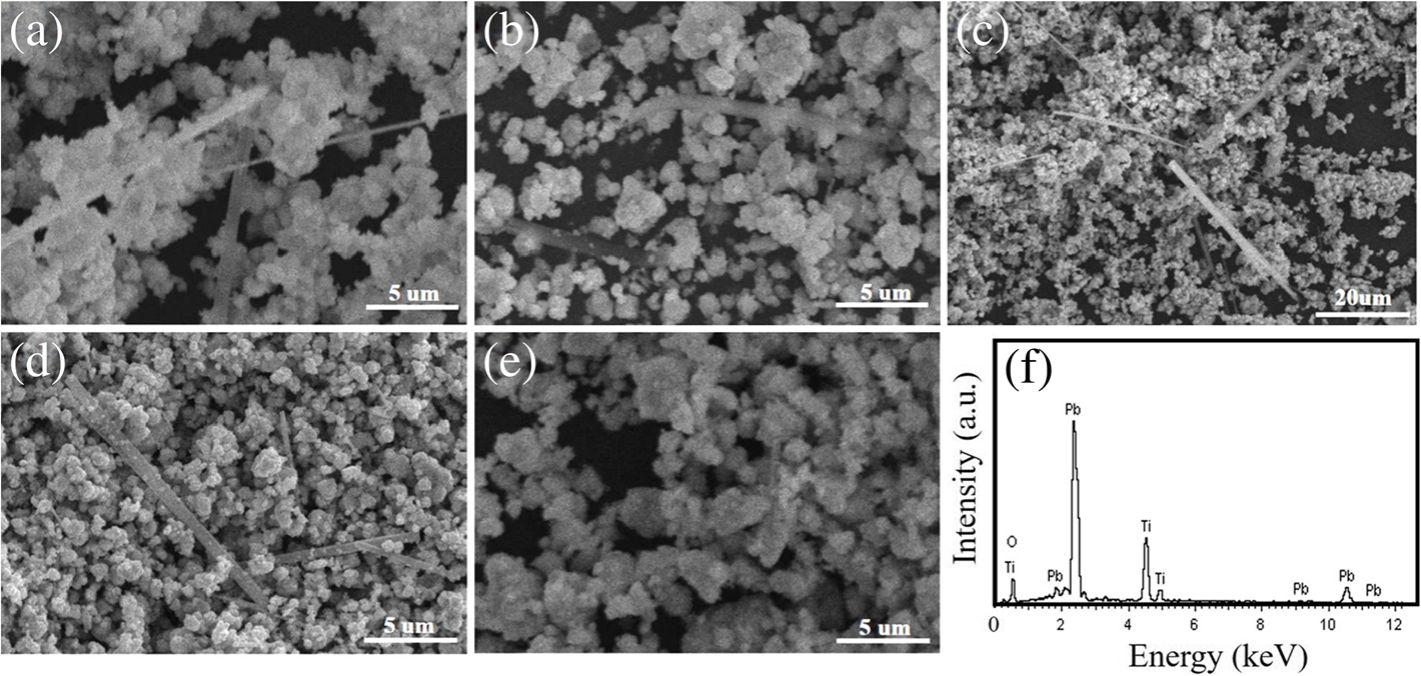
SEM images of the sphere-like PTO products synthesized by MSS method at 950 °C for 5 h with the molar ratios of PbC_2_O_4_:TiO_2_:NaCl:KCl equal to **a** 1:1:10:10, **b** 1:1:20:20, **c** 1:1:30:30, **d** 1:1:40:40, and **e** 1:1:60:60, respectively. **f** Typical EDS spectrum acquired from the sphere-like PTO products synthesized at 950 °C for 5 h with the molar ratio of PbC_2_O_4_:TiO_2_:NaCl:KCl equal to 1:1:10:10

XRD patterns of the PTO powders synthesized by MSS method at 950 °C for 5 h under different molten salt contents are shown in Fig. 1. It is observed that all the XRD diffraction peaks can be perfectly indexed to the tetragonal PTO (JCPDS No. 06–0452, *P*4*mm* space group, lattice constant *a* = 0.390 nm and *c* = 0.415 nm), and no other impurity phases are detected. Normally, the XRD pattern in the 2θ = 45° region is characteristic of the presence of either cubic or tetragonal perovskite structure. In this case, the splitting of cubic (200) into tetragonal (200) and (002) reflections at about 2θ = 45^°^ is clearly observed, indicating the formation of pure tetragonal ferroelectric phase. The lattice parameters (*a* and *c*) of the tetragonal PTO powders can be calculated by the following equation:

1 $$ \frac{1}{d^2}=\frac{h^2+{k}^2}{a^2}+\frac{l^2}{c^2} $$

where *d* is the interplanar spacing between the neighboring (*hkl*) planes, and *a* and *c* are the lattice parameters in the tetragonal phase structure. The lattice parameters *a* of the PTO powders calculated from XRD patterns were in the range of 0.3905–0.3911 nm, and *c* in the range of 0.4077–0.4089 nm. Details are presented in Table 1. The *c*/*a* ratio was in the range of 1.043–1.047 with an average value of 1.045, smaller than 1.064 for PTO single crystal. From the XRD patterns shown in Fig. 1, it can be observed that the phase structure of the PTO powders is not influenced by the molten salt content. All the PTO powders crystallized in a tetragonal phase structure with a space group of *P*4*mm*. Recently, the theoretical studies on the structural evolution of perovskite PTO from a 0D cluster to a 3D crystal by the CALYPSO (Crystal structure AnaLYsis by Particle Swarm Optimization) structure search method in conjunction with density functional theory calculations reveal that the ground state structure of PTO at ambient pressure is the *P*4*mm* phase and the quasi-planar structure of the PTO cluster and the 2D PTO double layer are also stable at ambient pressure [20]. The SEM-EDS examinations of the PTO powders are shown in Fig. 2. The SEM images shown in Fig. 2a–e reveal that the PTO powders mainly consist of many sphere-like nanoparticles except only a few rod-like particles. With increasing the molar ratio of PbC_2_O_4_:TiO_2_:NaCl:KCl from 1:1:10:10 to 1:1:60:60, the morphology of the PTO powders did not change apparently, as shown in Fig. 2a–e. That means, different amounts of the same molten salts only act as a reaction medium, they just have an influence on the diffusion rate of reaction ions. The eutectic NaCl-KCl molten salts (eutectic melting point 650 °C) provide a relatively low-temperature liquid-phase reaction environment, which aids the transportation of the reactant ions during the MSS process. It is reported that the reactant solubility in the molten salt plays an important role in MSS process, which affects the reaction rate and the morphology of the as-synthesized products critically [13]. In the present work, PbO is decomposed from PbC_2_O_4_ via the chemical reaction at temperatures of 400–500 °C [14]

2 $$ {\mathrm{PbC}}_2{\mathrm{O}}_4\to \mathrm{PbO}+\mathrm{CO}\uparrow +{\mathrm{CO}}_2\uparrow $$

which has higher solubility in the molten salt of NaCl-KCl (the solubility in NaCl-KCl salts is 30 μmol/g chlorides at 900 °C [21]) than TiO_2_ (which has very low solubility in alkali chlorides [22]). Therefore, the more soluble reactant PbO in the molten salt can diffuse onto the surface of sphere-like TiO_2_ precursor and react with it in situ to form sphere-like PTO powders by the solution-precipitation process. A typical EDS spectrum shown in Fig. 2f demonstrates that the sample is composed of Pb, Ti, and O elements, and the EDS analysis confirms the chemical composition is nearly similar to the nominal one.

**Table 1:** Lattice parameters of the spherical PbTiO_3_ powders synthesized by MSS method

Temperature (°C)	Reaction time (h)	Molar ratio of PbC_2_O_4_:TiO_2_:NaCl:KCl	Lattice parameters		
			*a* (nm)	*c* (nm)	*c*/*a*
950	5	1:1:10:10	0.3905	0.4085	1.046
950	5	1:1:20:20	0.3909	0.4089	1.046
950	5	1:1:30:30	0.3911	0.4083	1.044
950	5	1:1:40:40	0.3908	0.4091	1.047
950	5	1:1:60:60	0.3908	0.4077	1.043

### Phase Structure and Morphology of Rod-Like PTO Powders Synthesized by Template MSS Method

**Fig. 3:**
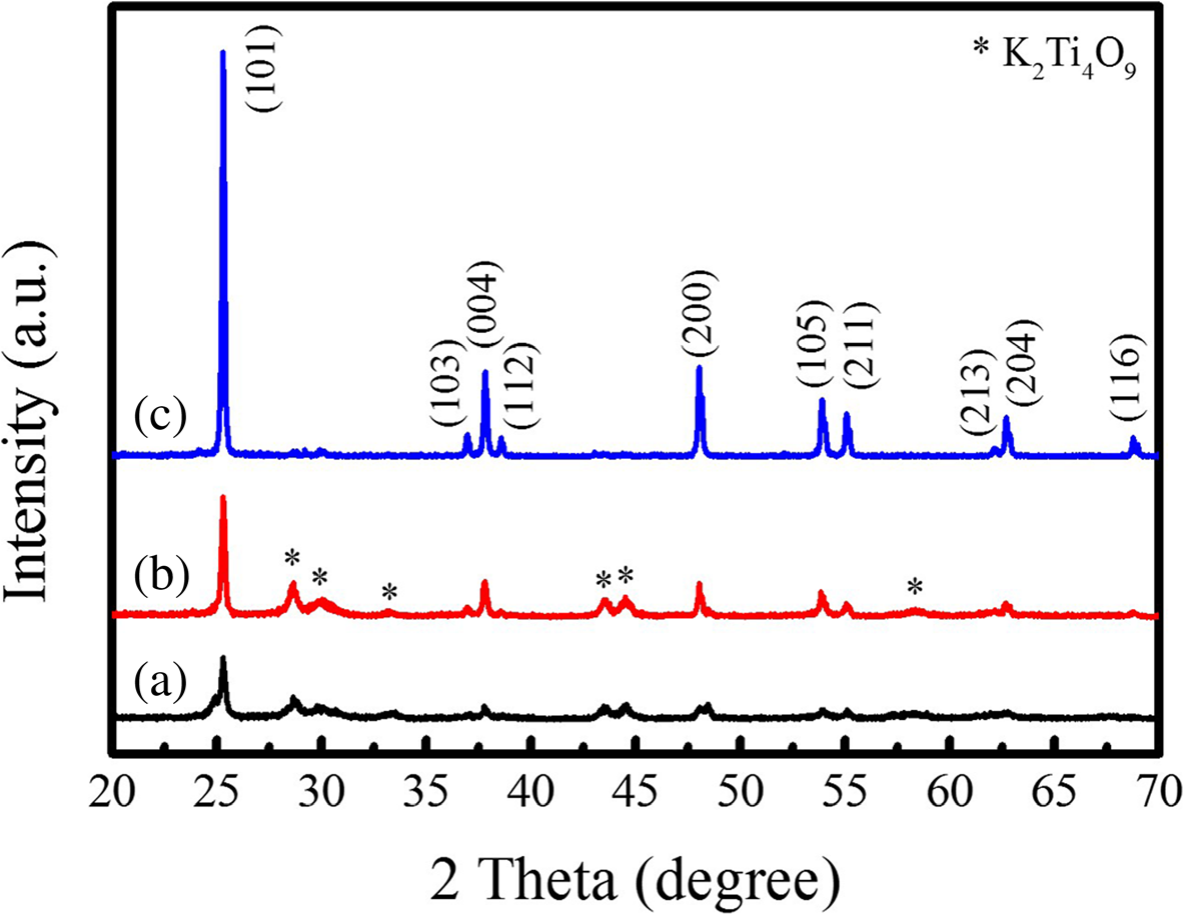
XRD patterns of the TiO_2_ templates annealed at (a) 600 °C, (b) 700 °C, and (c) 800 °C for 1 h

**Fig. 4:**
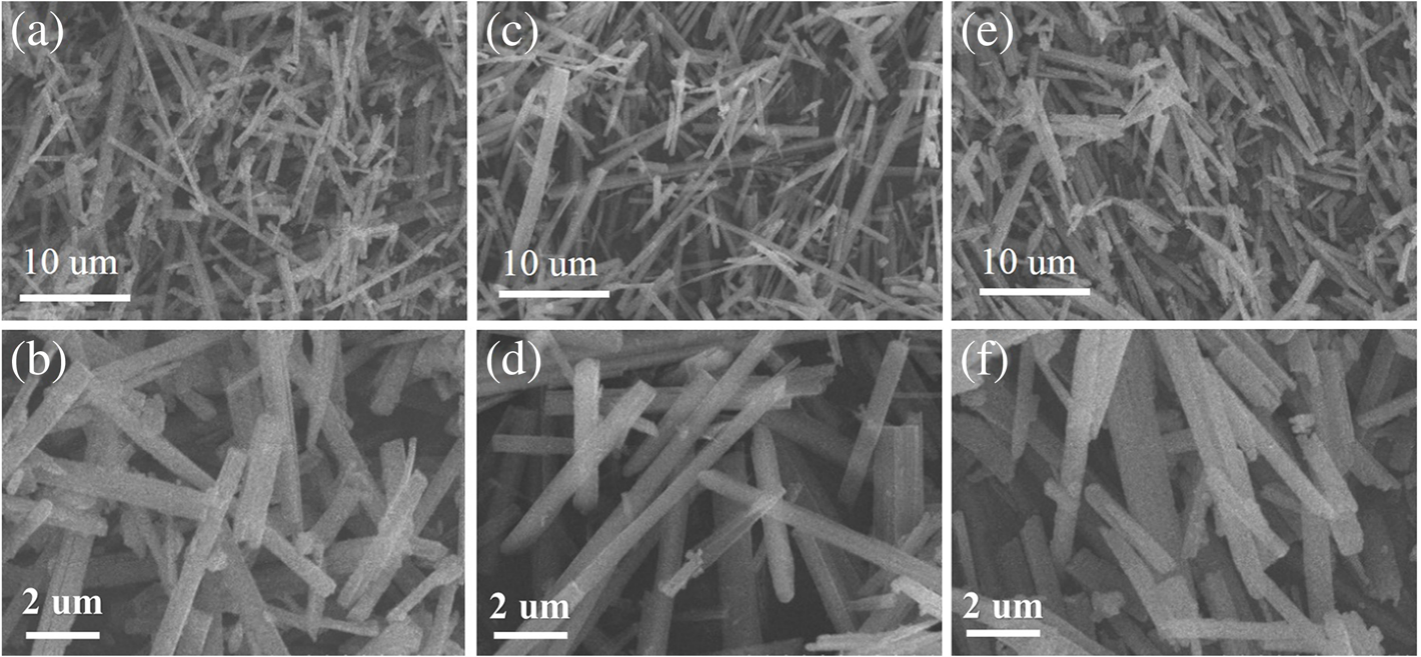
SEM images of the TiO_2_ templates annealed at **a**, **b** 600 °C; **c**, **d** 700 °C; and **e**, **f** 800 °C for 1 h

Before synthesizing the rod-like PTO powders by template MSS method, the phase structure and the morphology of the TiO_2_ templates were first investigated. Figure 3 demonstrates the XRD patterns of the TiO_2_ templates annealed at different temperatures for 1 h. It is observed that the predominant anatase phase of TiO_2_ was formed in the products after annealing at 600 °C (Fig. 3a) and 700 °C (Fig. 3b). However, a certain amount of K_2_Ti_4_O_9_ still retained in the products. The XRD diffraction peaks indicated by stars are originated from the K_2_Ti_4_O_9_ phase (ICDD no. 32-0861). With increasing the annealed temperature up to 800 °C (Fig. 3c), the impure K_2_Ti_4_O_9_ phase almost disappeared. All the XRD diffraction peaks shown in Fig. 3c can be well indexed to the anatase TiO_2_ (JCPDS No. 021–1272), indicating the formation of pure anatase phase TiO_2_. It was also noticed that the crystalline quality of the TiO_2_ template has much improved because the intensity of the (101) main diffraction peak has greatly increased. Figure 4 shows the SEM images of the TiO_2_ templates annealed at different temperatures. All the TiO_2_ templates exhibited rod-like morphology, and their average diameters varied from 490 nm to 570 nm and then 500 nm as the annealed temperatures increased while their average lengths were kept about 12.0 μm. The aspect ratios of the TiO_2_ templates annealed at 600 °C, 700 °C, and 800 °C were about 25, 23, and 24, respectively. The rod-like morphology of the annealed TiO_2_ templates is ascribed to the anisotropic growth of anatase phase structure in the annealed process. Based on the above experimental results, it can be concluded that the TiO_2_ templates with anatase phase annealed at 800 °C for 1 h have high crystallinity and rod-like morphology, which are apt to synthesize the rod-like PTO powders via template MSS method.

**Fig. 5:**
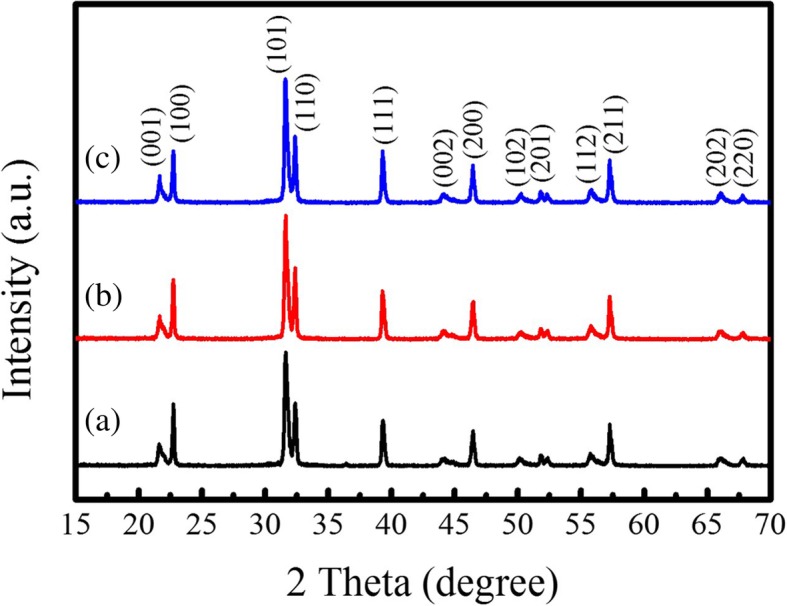
XRD patterns of the PTO powders synthesized via template MSS method with the molar ratio of PbC_2_O_4_:TiO_2_ (template):NaCl:KCl equal to 1:1:20:20 and at 800 °C for (a) 1 h, (b) 5 h, and (c) 10 h

**Fig. 6:**
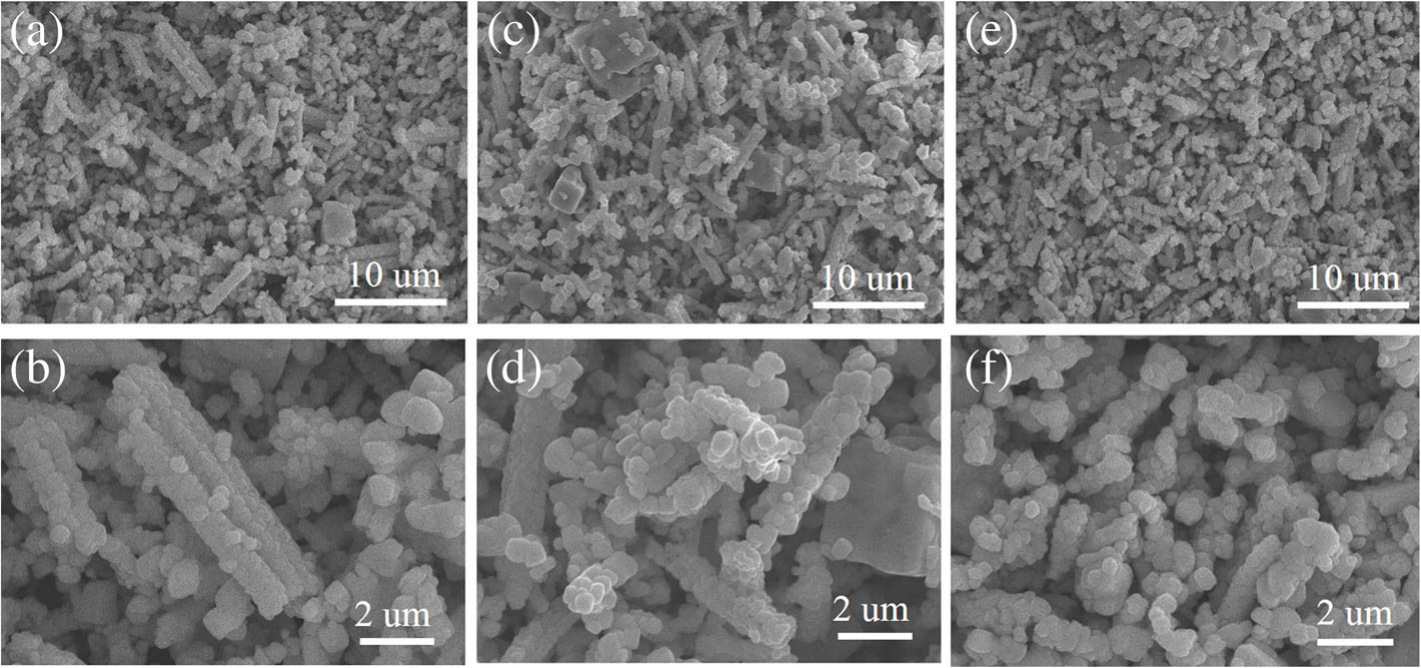
SEM images of the PTO products synthesized via template MSS method with the molar ratio of PbC_2_O_4_:TiO_2_ (template):NaCl:KCl equal to 1:1:20:20 and annealed at 800 °C for **a**, **b** 1 h; **c**, **d** 5 h; and **e**, **f** 10 h

Figure 5 shows the XRD patterns of the PTO powders synthesized by template MSS method at 800 °C for different reaction time, where the rod-like TiO_2_ templates (anatase phase annealed at 800 °C for 1 h) were used as the titanium source and the molar ratio of PbC_2_O_4_:TiO_2_ (template):NaCl:KCl was equal to 1:1:20:20. The XRD diffraction patterns reveal that all the PTO powders crystallize in a tetragonal phase structure (JCPDS No. 06–0452), and no other impurity phases are detected, illustrating the formation of pure tetragonal phase structure. The lattice parameters of the PTO powders were deduced from the XRD patterns, details are presented in Table 2. The corresponding SEM images of the PTO powders are shown in Fig. 6. As shown in Fig. 6a, the morphology of the PTO powders annealed at 800 °C for 1 h is a combination of a few rod-like and large amount of sphere-like PTO particles. The qualitative volume fraction of the rod-like PTO particles was only about 3%, and the rod-like PTO particles had an average diameter of about 860 nm and length of 4.50 μm. However, with increasing the reaction time from 1 h to 5 h, the volume fraction of the rod-like PTO particles was reduced to ~ 2.4% (Fig. 6c), and the rod-like PTO particles had an average diameter of about 930 nm and length of 6.0 μm. Further increasing the reaction time up to 10 h (Fig. 6e), the rod-like PTO particles were scarcely observed in the PTO products, whereas a large amount of sphere-like PTO particles were formed (see Fig. 6e-f). That means, prolonging the reaction time promotes the formation of spherical PTO particles whereas the formation of rod-like PTO particles is suppressed. The average particle size of the spherical PTO particles annealed at 800 °C for 10 h was about 550 nm (Fig. 6e), slightly larger than the diameter of the rod-like TiO_2_ template (480 nm) (Fig. 4e). The formation of large amount of spherical PTO particles in the products annealed at 800 °C for 10 h can be ascribed to that the rod-like TiO_2_ templates are broken into small spherical particles during the template MSS process, which react with the dissolved PbO (decomposed from PbC_2_O_4_) in the NaCl-KCl molten salt, forming spherical PTO powders via solution-precipitation mechanism. The broken trace of the TiO_2_ template was observed in Fig. 6b and d, where some spherical PTO particles were linked together to form the shape of sugar gourd string. It seems that the rod-like PTO powders are not successfully synthesized by template MSS method under low molten salt content.

**Table 2:** Lattice parameters of the PbTiO_3_ powders synthesized by template MSS method^*^

Temperature (°C)	Reaction time (h)	Molar ratio of PbC_2_O_4_:TiO_2_:NaCl:KCl in template MSS method					
		1:1:20:20			1:1:60:60		
		*a* (nm)	*c* (nm)	*c*/*a*	*a* (nm)	*c* (nm)	*c*/*a*
800	1	0.3906	0.4107	1.051	0.3906	0.4101	1.05
800	5	0.3908	0.4100	1.049	0.3907	0.4095	1.048
800	10	0.3907	0.4101	1.050	0.3905	0.4100	1.050

*The rod-like TiO_2_ templates annealed at 800 °C for 1 h with anatase phase structure were used as the titanium source in the template MSS process

**Fig. 7:**
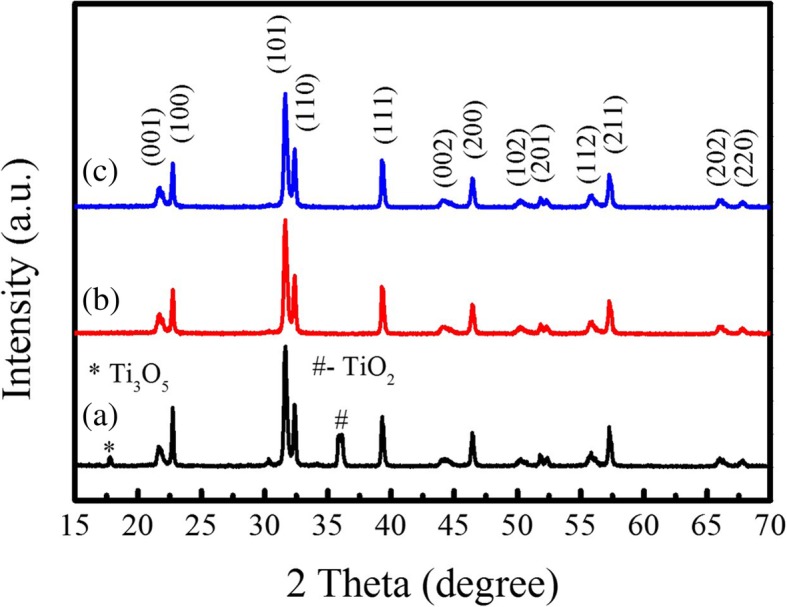
XRD patterns of the PTO powders synthesized via template MSS method with the molar ratio of PbC_2_O_4_: TiO_2_ (template):NaCl:KCl equal to 1:1:60:60 and annealed at 800 °C for (a) 1 h, (b) 5 h, and (c) 10 h

**Fig. 8:**
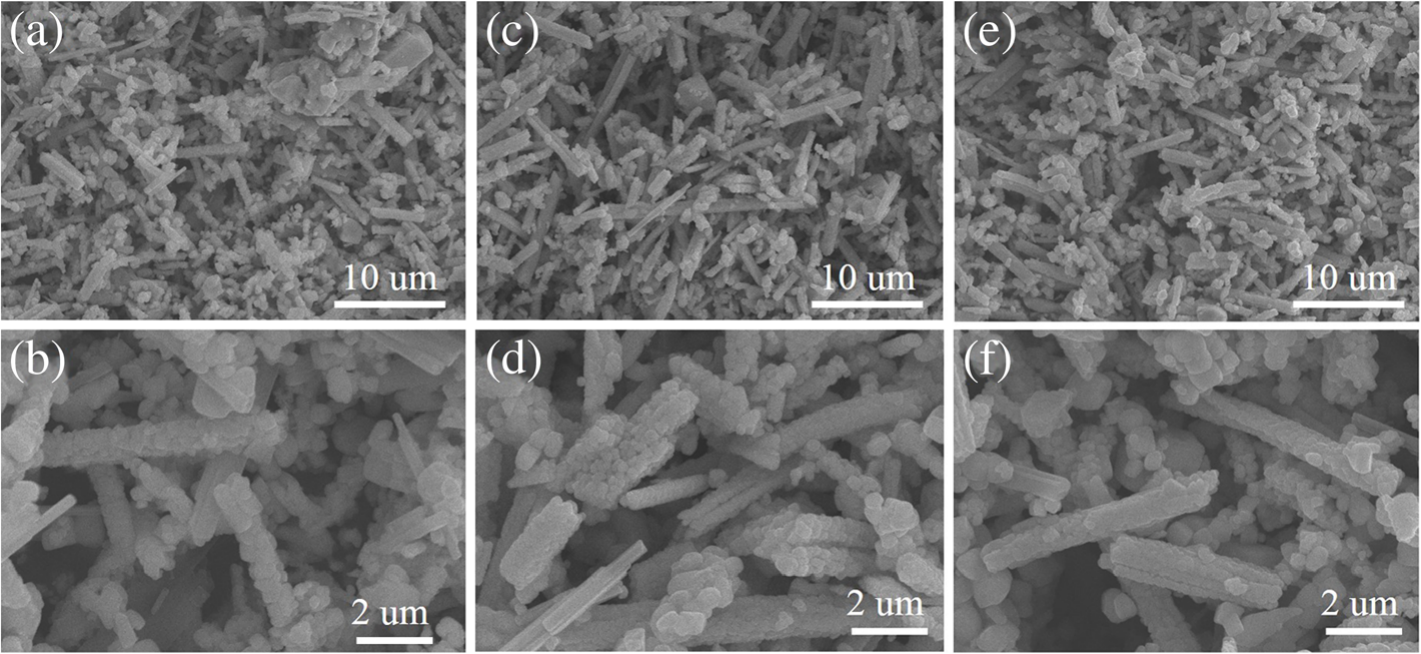
SEM images of the PTO powders synthesized via template MSS method with the molar ratio of PbC_2_O_4_:TiO_2_ (template):NaCl:KCl equal to 1:1:60:60 and at 800 °C for **a**, **b** 1 h; **c**, **d** 5 h; and **e**, **f** 10 h

It is known that in the MSS process, the grain grows through the melted salt fluxes at high temperatures, the molten salt content controls the grain growth rate and the morphology of the final products [23]. With increasing the molten salt content, larger amounts of molten salt liquid are formed at high temperature. Thus, the dissolved reaction ions (e.g., lead ions) are separated effectively in the molten salt liquid, and their concentrations require a longer time to achieve the saturation concentration. Therefore, the nucleation rate of PTO nanocrystallites at the surface of insoluble TiO_2_ template particles should be reduced. The formed PTO nanocrystallites have enough time to reunite them into rod-like PTO particles at the high molten salt content environment. That means, a high molten salt content environment is helpful to synthesize the rod-like PTO particles in the template MSS process. Therefore, we increased the molar ratio of PbC_2_O_4_:TiO_2_ (template):NaCl:KCl up to 1:1:60:60, and their mixtures were annealed at 800 °C for different hours. Figure 7 demonstrates the XRD patterns of the PTO powders synthesized at 800 °C by template MSS method under high molten salt content. It was found that the PTO powders annealed at 800 °C for 5 h (Fig. 7b) and 10 h (Fig. 7c) had pure tetragonal phase; however, the PTO powders annealed at 800 °C for 1 h (Fig. 7a) had a predominant tetragonal phase except the small impure phases of Ti_3_O_5_ and TiO_2_. The lattice constants *a* and *c* of the PTO powders annealed 800 °C for different hours were calculated and tabulated in Table 2. The *c*/*a* ratio was about 1.050. The surface morphologies of the corresponding PTO powders are shown in Fig. 8. It is observed in Fig. 8a that the PTO powders annealed at 800 °C for 1 h are composed of the rod- and sphere-like particles. The qualitative volume fraction of the rod-like particles estimated from SEM image was about 70%. The diameters of the rod-like particles varied from 480 nm to 1.50 μm while their lengths were in the range of 3.0–7.0 μm. The local enlarged SEM image shown in Fig. 8b reveals that the rod-like PTO powders are composed of very small PTO nanocrystallites, indicating the broken trace of the rod-like TiO_2_ templates during the template MSS process. With increasing the reaction time from 1 h to 5 h, the volume fraction of the rod-like PTO powders in the final product was increased up to ~ 97% (Fig. 8c). The length of the rod-like PTO powders was in the range of 7.0–10.0 μm. However, further increasing the reaction time up to 10 h (Fig. 8e), the volume fraction of the rod-like PTO particles in the final product was about 85%, and the length of the PTO rods was in the range of 3.5–6.5 μm. The diameter of the rod-like PTO powders was in the range of 970 nm–1.50 μm.

The formation of rod-like PTO powders under high molten salt content by template MSS method can be understood by the following process. In the template MSS process, PbC_2_O_4_ is first decomposed into PbO, CO, and CO_2_ in the temperature range of 400–500 °C, and PbO is dissolved into the molten salt liquid at 800 °C (its solubility in NaCl-KCl salts is 14 μmol/g chlorides at 800 °C [21]). The dissolved PbO diffuses onto the surface of the rod-like TiO_2_ template and reacts with TiO_2_ in situ to form PTO nanocrystallites via the chemical reaction at 800 °C

3 $$ \mathrm{PbO}+{\mathrm{TiO}}_2\to {\mathrm{PbTiO}}_3 $$

**Fig. 9:**
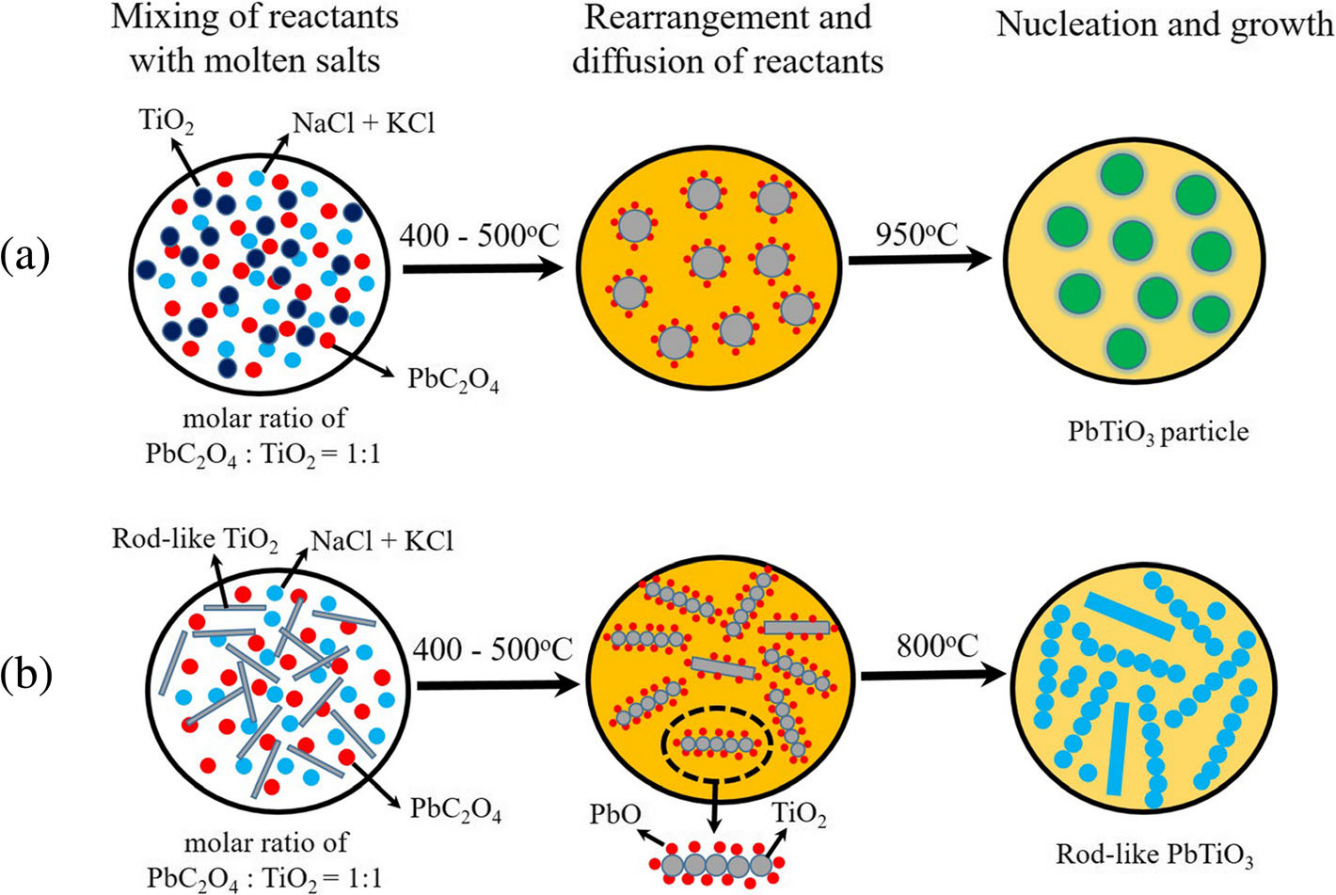
Schematic diagrams illustrating the formation of (a) PTO particles in the MSS process and (b) rod-like PTO powders in the template MSS process

Since the molten salt content is much high (the molar ratio of PbC_2_O_4_:TiO_2_ (template):NaCl:KCl equal to 1:1:60:60), so the dissolved lead ions are separated effectively in the molten salt liquid, its concentration needs a longer time to reach the saturation concentration. The rod-like TiO_2_ templates have very low solubility in NaCl-KCl salts, which are broken up into small TiO_2_ spherical particles at a high temperature under high molten salt content environment. Therefore, the dissolved PbO reacts with the broken TiO_2_ particles at their surfaces to form PTO nanocrystallites. These PTO nanocrystallites have enough time to reunite them into the rod-like PTO particles under high molten salt content environment. As shown in Fig. 8c, large-scale polycrystalline rod-like PTO powders with diameters in the range of 480 nm–1.50 μm and length up to 10 μm were synthesized. They are composed of small nanocrystallites as observed in Fig. 8d. The schematic diagrams illustrating the formation of PTO particles in the MSS process and rod-like PTO powders in the template MSS process are shown in Fig. 9. Our present work demonstrates that the molten salt content plays a critical role in forming the rod-like ABO_3_ compounds with cubic or pseudo-cubic crystal structure in the template MSS process. That is said under low molten salt content, the rod-like PTO powders cannot be synthesized even though the rod-like TiO_2_ templates are used in the template MSS process. The formation of the polycrystalline rod-like PTO powders in the shape of a sugar gourd string rather than single-crystalline PTO rods still needs further investigation.

### Dielectric Properties of Spherical and Rod-Like PTO Powders

**Fig. 10:**
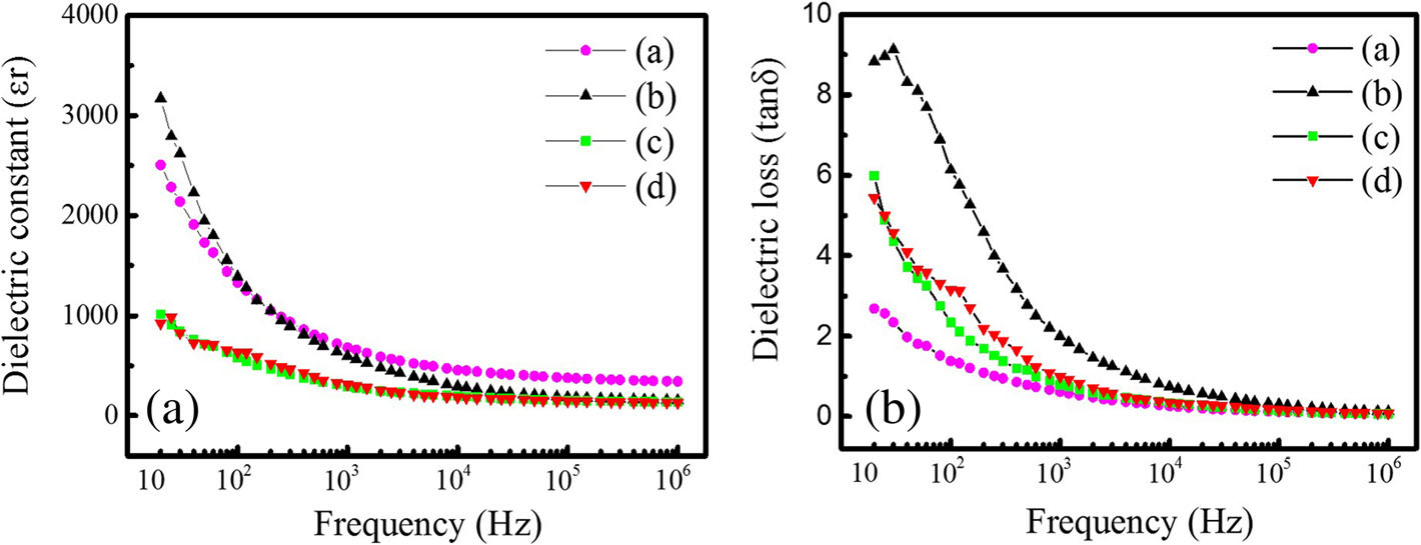
**a** Dielectric constants (*εr*) and **b** dielectric losses (tan*δ*) of the PTO powders synthesized by MSS method and template MSS method. Samples (a) and (b) were synthesized by MSS method at 950 °C for 5 h with the molar ratios of PbC_2_O_4_:TiO_2_:NaCl:KCl equal to 1:1:30:30 and 1:1:60:60, respectively. Samples (c) and (d) were synthesized by template MSS method with the molar ratio of PbC_2_O_4_:TiO_2_ (rod-like template):NaCl:KCl equal to 1:1:60:60 and annealed 800 °C for 5 h and 10 h, respectively

The dielectric constants (*εr*) and dielectric losses (tan*δ*) of the spherical and rod-like PTO powders synthesized by MSS and template MSS methods are shown in Fig. 10, which are measured at room temperature as a function of the frequency. Similar frequency-dependent dielectric behaviors are observed in the spherical and rod-like PTO powders. As shown in Fig. 10a, the sphere-like PTO powders (a) and (b) synthesized by MSS method at 950 °C for 5 h with the molar ratios of PbC_2_O_4_:TiO_2_:NaCl:KCl equal to (a) 1:1:30:30 and (b) 1:1:60:60, respectively, have much higher dielectric constants than the rod-like PTO powders (c) and (d) synthesized by template MSS method at 800 °C for (c) 5 h and (d) 10 h with the molar ratio of PbC_2_O_4_:TiO_2_ (template):NaCl:KCl equal to 1:1:60:60. It is noticed that the dielectric constants of the sphere-like PTO powders (a) and (b) have decreased fast (from ~ 3000 to ~ 700) in the frequency range below 10^3^ Hz, then reduced slowly with further increasing the frequency over 10^3^ Hz, and finally become a constant value of ~ 340 at a higher frequency over 10^5^ Hz. The fast decrease of the dielectric constant at lower frequencies is ascribed to the space charge polarization effect, which is correlated to the non-uniform charge accumulation at grain boundaries within the sphere-like PTO powders. The slow reduction of the dielectric constant is due to that the dipoles present in the PTO powders could not reorient themselves as fast as the frequency of an alternating electric field, resulting in a decrease of the dielectric constants [24]. In contrast, the rod-like PTO powders (c) and (d) synthesized by template MSS method exhibit a slight frequency-dependent dielectric behavior, their dielectric constants are slightly reduced with increasing frequency below 10^3^ Hz, and then become a constant value of ~ 140. It is observed in Fig. 10b that all the dielectric losses of the spherical and rod-like PTO powders are decreased with increasing frequency due to the existence of the space charge polarization in all the PTO powders. The dielectric loss of the PTO powder (b) has the highest value, which has reduced fast with increasing frequency below 10^5^ Hz, and then it becomes constant. The dielectric losses of the PTO powder (a) are reduced slowly with increasing frequency, which has the lowest value as compared with the other three PTO samples. The dielectric losses of the spherical PTO powders (c) and (d) synthesized by template MSS method exhibit very similar dielectric behavior, their dielectric losses are reduced slowly as the frequency increases. At room temperature, the dielectric constant and dielectric loss of the spherical PTO powders (a) measured at 10^6^ Hz were ~ 340 and 0.06, respectively. The corresponding values for the spherical PTO powders (b) were 155 and 0.12, 140 and 0.08 for the rod-like PTO powders (c), and 130 and 0.07 for the rod-like PTO powders (d). Therefore, the sphere-like PTO powders (a) have high dielectric constant and low dielectric loss, and these dielectric data are better than that reported previously for the PTO nanoparticles synthesized via sol-gel process and annealed at 600 °C for 6 h (the dielectric constant and dielectric loss at 10^6^ Hz were about 15 and 0.40) [25], and for the PTO nanoparticles synthesized by stearic acid gel method and annealed at 400 °C for 1 h (the dielectric constant and dielectric loss at 10^6^ Hz were about 50 and 0.002) [26]. Normally, to measure the dielectric properties of PbTiO_3_ ceramics prepared from the nanopowders synthesized by chemical methods such as the sol-gel method [27], hydrothermal method [28, 29], or by physical method such as high-energy ball milling technique [30], PbTiO_3_ powder samples are usually pressed into pellets under a hydraulic press (using 1 cm diameter die). For making dense PbTiO_3_ ceramics, the samples are needed to be sintered at high temperatures (e.g., 900 °C or 1000 °C for 2 h in air) followed by furnace cooling. Leonarska et al. [28] synthesized the PTO nanopowders at 490 K for 2 h by hydrothermal method and then prepared the PTO ceramics from the as-synthesized PTO nanopowders and sintered it at 1240 K for 1.5 h. They checked the impact of high-temperature process on the morphology or crystallization degree of the PTO ceramics by SEM observations and found that the PTO ceramics had slightly larger and rounded ceramic grains in comparison with the nanoparticles obtained directly from hydrothermal method. Similarly, Hu et al. [29] also reported the preparation of PTO nanoceramics (with grain sizes of ~ 200 nm) under sintering process (at 950 °C for 2 h in air) using the hydrothermal PTO nanopowders (with average grain size of ~ 100 nm) as the raw materials. This result indicates the sintering process of the hydrothermal PTO nanopowders can increase the grain size. Kong et al. [30] prepared the PTO nanopowders (with average grain size of ~ 10 nm) by high-energy ball milling technique, and pressed them directly into green pellets and sintered at 1100 °C for 1 h. Crack-free PTO ceramics with 95% of the theoretical density were successfully obtained. SEM images revealed that the average grain size of the sintered samples were < 1.5 μm. In the present work, we have prepared dense PbTiO_3_ ceramic samples under a sintering process (at 1150 °C for 2 h) by using the as-synthesized sphere-like PTO powders via MSS method and the as-synthesized rod-like PTO powders via template MSS method. The high-temperature process has improved the crystallized quality and the grain sizes of the PTO powders but has few effects on the morphology. The best dielectric properties of the PTO ceramics prepared the as-synthesized spherical PTO powders by MSS method are attributed to their larger average particle size (~ 2.0 μm).

## Conclusions

Both sphere- and rod-like PTO powders were synthesized by MSS and template MSS methods, respectively. XRD patterns reveal that all the PTO powders are crystallized in a tetragonal phase structure. SEM images demonstrated that increasing the molar ratio of PbC_2_O_4_:TiO_2_:NaCl:KCl from 1:1:10:10 to 1:1:60:60 in the MSS process had little effect on the sphere-like morphology of the PTO powders synthesized by MSS method. Large-scale polycrystalline rod-like PTO powders with length up to 10 μm and diameters in the range of 480 nm–1.50 μm were successfully synthesized by template MSS method at 800 °C for 5 h, where the rod-like anatase TiO_2_ precursors were used as a titanium source and the molar ratio of PbC_2_O_4_:TiO_2_:NaCl:KCl was equal to 1:1:60:60. It is found that under low molten salt content, extending the reaction time promoted the formation of sphere-like PTO particles whereas the formation of rod-like PTO particles was suppressed. In addition, the rod-like PTO powders cannot be synthesized even if the rod-like TiO_2_ templates are used. Dielectric measurements demonstrated that the dielectric constants of the sphere-like PTO powders synthesized by MSS method decreased fast from ~ 3000 to ~ 700 at low frequencies below 10^3^ Hz, and at high frequencies over 10^5^ Hz they became a constant value of ~ 340. The fast decrease of the dielectric constant at low frequencies is ascribed to the space charge polarization due to the non-uniform charges accumulated within the PTO powders. The rod-like PTO powders synthesized by template MSS method exhibited slight frequency-dependent dielectric behavior, their dielectric constants decreased slowly at the frequencies below 10^3^ Hz and then remained a constant value of ~ 140 as the frequency increased up to 10^6^ Hz. At room temperature, dielectric constant and dielectric loss (measured at 10^6^ Hz) of the sphere-like PTO powders synthesized by MSS method at 950 °C for 5 h with low molten salt content (the molar ratio of PbC_2_O_4_:TiO_2_:NaCl:KCl equal to 1:1:30:30) were 340 and 0.06, respectively, and the corresponding values were 155 and 0.12 for the sphere-like PTO powders synthesized by MSS method with high molten salt content (the molar ratio of PbC_2_O_4_:TiO_2_:NaCl:KCl equal to 1:1:60:60). The dielectric constant and dielectric loss for the rod-like PTO powders synthesized by template MSS method at 800 °C for 5 h and 10 h under high molten salt content (the molar ratio of PbC_2_O_4_:TiO_2_ (rod-like template):NaCl:KCl equal to 1:1:60:60) were 140 and 0.08, and 130 and 0.07, respectively. The higher dielectric constant and lower dielectric loss of the sphere-like PTO powders synthesized at 950 °C for 5 h by MSS method with the molar ratio of PbC_2_O_4_:TiO_2_:NaCl:KCl equal to 1:1:30:30 are ascribed to their large average particle size (~ 2.0 μm), which have promising applications in multilayer capacitors and resonators.

## Abbreviations

CALYPSO: Crystal Structure AnaLYsis by Particle Swarm Optimization
EDS: Energy Dispersive Spectroscopy
MSS: Molten Salt Synthesis
NP-9: Polyoxyethylene (9) Nonylphenyl Ether
PTO: PbTiO_3_
SEM: Scanning Electron Microscopy
XRD: X-ray Diffraction

## References

[1] Peña AA, Fierro JLG (2001). Chemical structures and performance of perovskite oxides. Chem Rev.

[2] Li LH, Deng JX, Chen J, Xing XR (2016). Topochemical molten salt synthesis for functional perovskite compounds. Chem Sci.

[3] Xue PJ, Wu H, Lu Y, Zhu XH (2018). Recent progress in molten salt synthesis of low-dimensional perovskite oxide nanostructures, structural characterization, physical properties and applications. J Mater Sci Technol.

[4] Jaffe B, Cook WR, Jaffe H (1971). Piezoelectric deramics.

[5] Selbach SM, Wang GZ, Einarsrud MA, Grande T (2007). Decomposition and crystallization of a sol–del-derived PbTiO_3_ precursor. J Am Ceram Soc.

[6] Udomporn A, Ananta S (2008). Effect of calcinations condition on phase formation and particle size of lead titanate powders synthesized by the solid-state reaction. Mater Lett.

[7] Rodriguez-Aranda MC, Calderon-Pinar F, Hernandez-Landaverde MA, Heiras J, Zamorano-Ulloa R, Ramirez-Rosales D, Yanez-Limon JM (2016) Photoluminescence of sol-gel synthesized PZT powders. J Lumin 179:280–286

[8] Lee CY, Tai NH, Sheu HS, Chiu HT, Hsieh SH (2006). The formation of perovskite PbTiO_3_ powders by sol–gel process. Mater Chem Phys.

[9] Petersona R, Siamovic B (1999). Effect of processing parameters on the morphology of hydrothermally derived PbTiO_3_ powders. J Am Ceram Soc.

[10] Chen X, Fan H, Liu L (2005). Synthesis and crystallization behavior of lead titanate from oxide precursors by a hydrothermal route. J Cryst Growth.

[11] Paris EC, Leite ER, Longo E, Varela JA (1998). Synthesis of PbTiO_3_ by use of polymeric precursors. Mater Lett.

[12] Fang J, Wang J, Ng SC, Chew CH, Gan LM (1999). Preparation and characterization of ultrafine lead titanate (PbTiO_3_) powders. J Mater Sci.

[13] Kimura T, Sikalidis C (2011). Molten salt synthesis of ceramic powders. Advances in ceramics synthesis and characterization, processing and specific applications.

[14] Cai ZY, Xin XR, Liu GR, Yu RB (2006). Synthesis of PbTiO_3_ powder by molten salt method and its characteristics. Acta Metall Sin.

[15] Cai ZY, Xing XR, Yu RB, Sun XY, Liu GR (2007). Morphology-controlled synthesis of lead titanate powders. Inorg Chem.

[16] Rørvik PM, Lyngdal T, Sæterli R, van HATJ, Holmestad R, Grande T, Einarsrud MA (2008). Influence of volatile chlorides on the molten salt synthesis of ternary oxide nanorods and nanoparticles. Inorg Chem.

[17] Deng Y, Wang JL, Zhu KR, Zhang MS, Hong JM, Gu QR, Yin Z (2005). Synthesis and characterization of single-crystal PbTiO_3_ nanorods. Mater Lett.

[18] Ma S, Fuh JYH, Zhang YF, Lu L (2010). Synthesis of anisotropic lead titanate powders for templated grain growth of textured piezoelectric ceramics. Surf Rev Lett.

[19] Hayashi Y, Kimura T, Yamaguchi T (1986). Preparation of rod-shaped BaTiO_3_ powder. J Mater Sci.

[20] Lu C, Wang JJ, Wang P, Xia XX, Jin YY, Li PF, Bao G (2017). New insight into the structural evolution of PbTiO_3_: an unbiased structure search. Phys Chem Chem Phys.

[21] Li CC, Chiu CC, Desu SB (1992). Formation of lead niobates in molten salt systems. J Am Ceram Soc.

[22] Anikin IN, Naumova II, Rumyantseva GV (1965). Solubility of titanium dioxide in molten salts and crystallization of rutile. Sov Phys Crystallogr (Engl Trans).

[23] Yoon KH, Cho YS, Kang DH (1998). Molten salt synthesis of lead-based relaxors. J Mater Sci.

[24] Selvi KT, Alamelumangai K, Priya M, Rathnakumari M, Kumar PS, Sagadevan S (2016). Studies on synthesis, structural, surface morphological and electrical properties of Pr_6_O_11_–MgO nanocomposite. J Mater Sci Mater Electron.

[25] Sagadevan S, Das I, Podder J (2016). Synthesis of lead titanate nanoparticles via sol–gel technique and its characterization. J Mater Sci Mater Electron.

[26] Ruan SP, Wang J, Zhang L, Liu YG, Ma J, Xuan L, Xu BK (2003). Synthesis and dielectric properties of nanocrystalline PbTiO_3_. J Funct Mater Dev.

[27] Pavithra C, Madhuri W (2018). Dielectric, piezo and ferroelectric properties of microwave sintered PbTiO_3_ synthesized by sol–gel method. J Sol-Gel Sci Technol.

[28] Leonarska A, Ujma Z, Molak A (2014). Nano-size grain powders and ceramics of PbTiO_3_ obtained by the hydrothermal method and their electrical properties. Ferroelectrics.

[29] Hu YM, Gu HS, Chen WP, Wang Y (2010). Preparation of PbTiO_3_ nanoceramics based on hydrothermal nanopowders and characterization of their electrical properties. Mater Chem Phys.

[30] Kong LB, Zhu W, Tan OK (2000). PbTiO_3_ ceramics derived from high-energy ball milled nano-sized powders. J Mater Sci Lett.

